# 1,5-Bis[(2-meth­oxy­eth­oxy)meth­yl]-1,5-naphthyridine-4,8(1*H*,5*H*)-dione

**DOI:** 10.1107/S1600536811054547

**Published:** 2012-01-07

**Authors:** Kunyan Wang, Chen Chen, Peng Jiang, Lu Shi, Hong-Jun Zhu

**Affiliations:** a Department of Applied Chemistry, College of Science, Nanjing University of Technology, Nanjing 210009, People’s Republic of China

## Abstract

The complete mol­ecule of the title compound, C_16_H_22_N_2_O_6_, is generated by crystallographic inversion symmetry. The conformation of the N—C—O—C fragment of the side chain is approximately gauche [torsion angle = −74.84 (17)°]. In the crystal, weak C—H⋯O inter­actions link the mol­ecules.

## Related literature

The background to the applications of the title compound, see: Shan *et al.* (2005 ▶). For the synthesis, see: Toshihiro *et al.* (2002 ▶). For standard bond lengths, see: Allen *et al.* (1987 ▶).
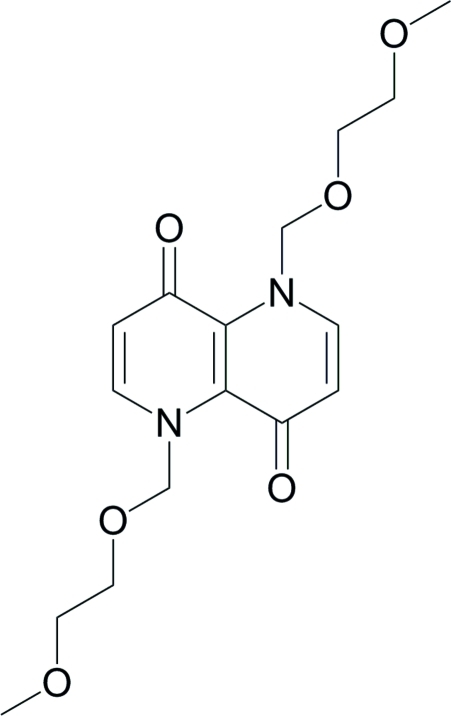


## Experimental

### 

#### Crystal data

C_16_H_22_N_2_O_6_
*M* *_r_* = 338.36
Monoclinic, 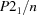

*a* = 7.1610 (14) Å
*b* = 11.497 (2) Å
*c* = 10.734 (2) Å
β = 105.45 (3)°
*V* = 851.8 (3) Å^3^
*Z* = 2
Mo *K*α radiation
μ = 0.10 mm^−1^
*T* = 293 K
0.30 × 0.20 × 0.10 mm

#### Data collection

Enraf–Nonius CAD-4 diffractometer
Absorption correction: ψ scan (North *et al.*, 1968 ▶) *T*_min_ = 0.970, *T*_max_ = 0.990
3261 measured reflections
1549 independent reflections
1246 reflections with *I* > 2σ(*I*)
*R*_int_ = 0.047
3 standard reflections every 200 reflections intensity decay: 1%

#### Refinement

*R*[*F* ^2^ > 2σ(*F* ^2^)] = 0.045
*wR*(*F* ^2^) = 0.140
*S* = 1.01
1549 reflections
110 parameters
H-atom parameters constrained
Δρ_max_ = 0.24 e Å^−3^
Δρ_min_ = −0.17 e Å^−3^

### 

Data collection: *CAD-4 EXPRESS* (Enraf–Nonius, 1994 ▶); cell refinement: *CAD-4 EXPRESS*; data reduction: *XCAD4* (Harms & Wocadlo, 1995 ▶); program(s) used to solve structure: *SHELXS97* (Sheldrick, 2008 ▶); program(s) used to refine structure: *SHELXL97* (Sheldrick, 2008 ▶); molecular graphics: *SHELXTL* (Sheldrick, 2008 ▶); software used to prepare material for publication: *SHELXTL*.

## Supplementary Material

Crystal structure: contains datablock(s) I, global. DOI: 10.1107/S1600536811054547/hb6550sup1.cif

Structure factors: contains datablock(s) I. DOI: 10.1107/S1600536811054547/hb6550Isup2.hkl

Supplementary material file. DOI: 10.1107/S1600536811054547/hb6550Isup3.cml

Additional supplementary materials:  crystallographic information; 3D view; checkCIF report

## Figures and Tables

**Table 1:** Hydrogen-bond geometry (Å, °)

*D*—H⋯*A*	*D*—H	H⋯*A*	*D*⋯*A*	*D*—H⋯*A*
C5—H5*A*⋯O3^i^	0.93	2.45	3.264 (2)	147
C6—H6*A*⋯O1^ii^	0.93	2.58	3.397 (2)	147

Symmetry codes: (i) 
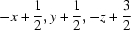
; (ii) 
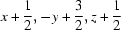
.

**Fig. 1.**
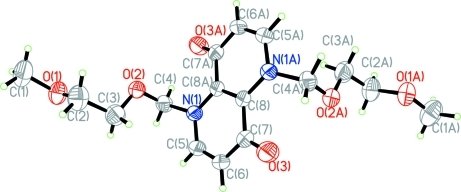
The molecular structure of (I), with the atom-numbering scheme. Displacement ellipsoids are drawn at the 50% probability level.

**Fig. 2.**
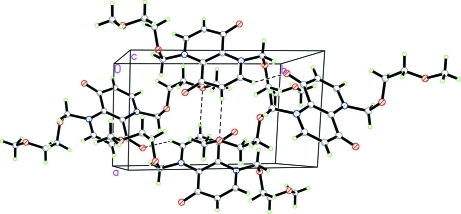
A packing diagram of (I). Hydrogen bonds are shown as dashed lines.

## supplementary crystallographic information

### Experimental

The title compound was prepared by a method reported in literature (Toshihiro *et al.*, 2002). Colourless blocks were obtained by dissolving it (0.5 g) in methanol (50 ml) and evaporating the solvent slowly at room temperature for about 30 d.

### Refinement

H atoms were positioned geometrically and refined as riding groups, with C—H = 0.93 Å for aromatic H, and constrained to ride on their parent atoms, with *U*_iso_(H) = *xU*_eq_(C), where *x* = 1.2 for aromatic H, and *x* = 1.5 for other H.

### Crystal data

**Table:**

C_16_H_22_N_2_O_6_	*F*(000) = 360
*M**_r_* = 338.36	*D*_x_ = 1.319 Mg m^−^^3^
Monoclinic, *P*2_1_/*n*	Melting point: 365 K
Hall symbol: -P 2yn	Mo *K*α radiation, λ = 0.71073 Å
*a* = 7.1610 (14) Å	Cell parameters from 25 reflections
*b* = 11.497 (2) Å	θ = 9–13°
*c* = 10.734 (2) Å	µ = 0.10 mm^−^^1^
β = 105.45 (3)°	*T* = 293 K
*V* = 851.8 (3) Å^3^	Block, colourless
*Z* = 2	0.30 × 0.20 × 0.10 mm

### Data collection

**Table:**

Enraf–Nonius CAD-4 diffractometer	1246 reflections with *I* > 2σ(*I*)
Radiation source: fine-focus sealed tube	*R*_int_ = 0.047
graphite	θ_max_ = 25.3°, θ_min_ = 2.7°
ω/2θ scans	*h* = 0→8
Absorption correction: ψ scan (North *et al.*, 1968)	*k* = −13→13
*T*_min_ = 0.970, *T*_max_ = 0.990	*l* = −12→12
3261 measured reflections	3 standard reflections every 200 reflections
1549 independent reflections	intensity decay: 1%

### Refinement

**Table:**

Refinement on *F*^2^	Secondary atom site location: difference Fourier map
Least-squares matrix: full	Hydrogen site location: inferred from neighbouring sites
*R*[*F*^2^ > 2σ(*F*^2^)] = 0.045	H-atom parameters constrained
*wR*(*F*^2^) = 0.140	*w* = 1/[σ^2^(*F*_o_^2^) + (0.1*P*)^2^ + 0.026*P*] where *P* = (*F*_o_^2^ + 2*F*_c_^2^)/3
*S* = 1.01	(Δ/σ)_max_ < 0.001
1549 reflections	Δρ_max_ = 0.24 e Å^−^^3^
110 parameters	Δρ_min_ = −0.17 e Å^−^^3^
0 restraints	Extinction correction: *SHELXL97* (Sheldrick, 2008), Fc^*^=kFc[1+0.001xFc^2^λ^3^/sin(2θ)]^-1/4^
Primary atom site location: structure-invariant direct methods	Extinction coefficient: 0.30 (2)

### Special details

**Table:**

Geometry. All e.s.d.'s (except the e.s.d. in the dihedral angle between two l.s. planes) are estimated using the full covariance matrix. The cell e.s.d.'s are taken into account individually in the estimation of e.s.d.'s in distances, angles and torsion angles; correlations between e.s.d.'s in cell parameters are only used when they are defined by crystal symmetry. An approximate (isotropic) treatment of cell e.s.d.'s is used for estimating e.s.d.'s involving l.s. planes.
Refinement. Refinement of *F*^2^ against ALL reflections. The weighted *R*-factor *wR* and goodness of fit *S* are based on *F*^2^, conventional *R*-factors *R* are based on *F*, with *F* set to zero for negative *F*^2^. The threshold expression of *F*^2^ > σ(*F*^2^) is used only for calculating *R*-factors(gt) *etc*. and is not relevant to the choice of reflections for refinement. *R*-factors based on *F*^2^ are statistically about twice as large as those based on *F*, and *R*- factors based on ALL data will be even larger.

### Fractional atomic coordinates and isotropic or equivalent isotropic displacement parameters (Å^2^)

**Table:**

	*x*	*y*	*z*	*U*_iso_*/*U*_eq_	
N1	0.03696 (18)	0.64646 (11)	0.57178 (12)	0.0421 (4)	
O1	0.25472 (18)	1.00854 (10)	0.36603 (13)	0.0584 (4)	
C1	0.2858 (3)	1.0712 (2)	0.2607 (2)	0.0781 (7)	
H1A	0.2389	1.1492	0.2623	0.117*	
H1B	0.2177	1.0341	0.1815	0.117*	
H1C	0.4219	1.0729	0.2665	0.117*	
O2	0.06007 (17)	0.80910 (9)	0.43882 (11)	0.0532 (4)	
C5	0.1950 (2)	0.62630 (14)	0.67339 (15)	0.0479 (5)	
H5A	0.2437	0.6875	0.7292	0.057*	
C2	0.3185 (3)	0.89321 (17)	0.3692 (2)	0.0658 (6)	
H2B	0.4581	0.8917	0.3836	0.079*	
H2C	0.2603	0.8560	0.2869	0.079*	
C3	0.2645 (3)	0.82888 (15)	0.4743 (2)	0.0639 (6)	
H3A	0.3325	0.7551	0.4890	0.077*	
H3B	0.3014	0.8736	0.5537	0.077*	
O3	0.32189 (18)	0.33513 (10)	0.62397 (14)	0.0672 (5)	
C4	−0.0118 (2)	0.77036 (13)	0.54011 (17)	0.0494 (5)	
H4A	−0.1514	0.7797	0.5165	0.059*	
H4B	0.0419	0.8177	0.6160	0.059*	
C6	0.2837 (2)	0.52236 (14)	0.69674 (16)	0.0489 (5)	
H6A	0.3860	0.5132	0.7704	0.059*	
C7	0.2259 (2)	0.42641 (13)	0.61247 (15)	0.0449 (4)	
C8	0.0437 (2)	0.44520 (12)	0.50952 (13)	0.0380 (4)	

### Atomic displacement parameters (Å^2^)

**Table:**

	*U*^11^	*U*^22^	*U*^33^	*U*^12^	*U*^13^	*U*^23^
N1	0.0493 (7)	0.0351 (7)	0.0376 (7)	−0.0011 (5)	0.0041 (5)	−0.0016 (5)
O1	0.0678 (8)	0.0436 (7)	0.0690 (9)	0.0043 (5)	0.0273 (7)	0.0064 (5)
C1	0.0727 (13)	0.0781 (15)	0.0928 (16)	0.0075 (11)	0.0385 (12)	0.0289 (12)
O2	0.0602 (8)	0.0402 (6)	0.0513 (7)	−0.0052 (5)	0.0012 (6)	0.0059 (5)
C5	0.0557 (9)	0.0440 (9)	0.0374 (9)	−0.0068 (7)	0.0009 (7)	−0.0030 (6)
C2	0.0697 (11)	0.0493 (11)	0.0836 (14)	0.0087 (9)	0.0291 (10)	0.0052 (9)
C3	0.0577 (11)	0.0523 (11)	0.0756 (13)	0.0029 (8)	0.0071 (9)	0.0127 (9)
O3	0.0602 (8)	0.0429 (7)	0.0812 (10)	0.0086 (5)	−0.0114 (7)	0.0018 (6)
C4	0.0565 (9)	0.0341 (8)	0.0532 (10)	0.0014 (7)	0.0066 (7)	−0.0044 (7)
C6	0.0490 (9)	0.0464 (9)	0.0413 (9)	−0.0051 (7)	−0.0054 (7)	0.0051 (7)
C7	0.0459 (9)	0.0396 (8)	0.0446 (9)	0.0003 (7)	0.0040 (7)	0.0083 (7)
C8	0.0442 (8)	0.0341 (8)	0.0346 (8)	−0.0035 (6)	0.0086 (6)	0.0034 (6)

### Geometric parameters (Å, °)

**Table:**

N1—C5	1.366 (2)	C2—H2B	0.9700
N1—C8^i^	1.3919 (19)	C2—H2C	0.9700
N1—C4	1.4843 (19)	C3—H3A	0.9700
O1—C2	1.400 (2)	C3—H3B	0.9700
O1—C1	1.407 (2)	O3—C7	1.2426 (18)
C1—H1A	0.9600	C4—H4A	0.9700
C1—H1B	0.9600	C4—H4B	0.9700
C1—H1C	0.9600	C6—C7	1.417 (2)
O2—C4	1.394 (2)	C6—H6A	0.9300
O2—C3	1.429 (2)	C7—C8	1.484 (2)
C5—C6	1.345 (2)	C8—N1^i^	1.3919 (19)
C5—H5A	0.9300	C8—C8^i^	1.398 (3)
C2—C3	1.484 (3)		
			
C5—N1—C8^i^	119.30 (13)	O2—C3—H3A	109.8
C5—N1—C4	116.10 (13)	C2—C3—H3A	109.8
C8^i^—N1—C4	123.37 (13)	O2—C3—H3B	109.8
C2—O1—C1	112.60 (16)	C2—C3—H3B	109.8
O1—C1—H1A	109.5	H3A—C3—H3B	108.2
O1—C1—H1B	109.5	O2—C4—N1	111.85 (13)
H1A—C1—H1B	109.5	O2—C4—H4A	109.2
O1—C1—H1C	109.5	N1—C4—H4A	109.2
H1A—C1—H1C	109.5	O2—C4—H4B	109.2
H1B—C1—H1C	109.5	N1—C4—H4B	109.2
C4—O2—C3	114.06 (14)	H4A—C4—H4B	107.9
C6—C5—N1	123.30 (15)	C5—C6—C7	121.98 (14)
C6—C5—H5A	118.3	C5—C6—H6A	119.0
N1—C5—H5A	118.3	C7—C6—H6A	119.0
O1—C2—C3	109.96 (17)	O3—C7—C6	122.30 (14)
O1—C2—H2B	109.7	O3—C7—C8	123.39 (14)
C3—C2—H2B	109.7	C6—C7—C8	114.30 (13)
O1—C2—H2C	109.7	N1^i^—C8—C8^i^	119.73 (16)
C3—C2—H2C	109.7	N1^i^—C8—C7	119.50 (13)
H2B—C2—H2C	108.2	C8^i^—C8—C7	120.73 (16)
O2—C3—C2	109.45 (16)		
			
C8^i^—N1—C5—C6	2.9 (3)	N1—C5—C6—C7	3.7 (3)
C4—N1—C5—C6	−164.78 (16)	C5—C6—C7—O3	170.47 (16)
C1—O1—C2—C3	−174.45 (17)	C5—C6—C7—C8	−8.8 (2)
C4—O2—C3—C2	−167.81 (14)	O3—C7—C8—N1^i^	6.6 (2)
O1—C2—C3—O2	72.0 (2)	C6—C7—C8—N1^i^	−174.16 (14)
C3—O2—C4—N1	−74.84 (17)	O3—C7—C8—C8^i^	−171.19 (18)
C5—N1—C4—O2	97.84 (16)	C6—C7—C8—C8^i^	8.1 (2)
C8^i^—N1—C4—O2	−69.31 (19)		

Symmetry codes: (i) −*x*, −*y*+1, −*z*+1.

### Hydrogen-bond geometry (Å, °)

**Table:**

*D*—H···*A*	*D*—H	H···*A*	*D*···*A*	*D*—H···*A*
C5—H5A···O3^ii^	0.93	2.45	3.264 (2)	147
C6—H6A···O1^iii^	0.93	2.58	3.397 (2)	147

Symmetry codes: (ii) −*x*+1/2, *y*+1/2, −*z*+3/2; (iii) *x*+1/2, −*y*+3/2, *z*+1/2.
